# Focused transesophageal echocardiography for emergency physicians—description and results from simulation training of a structured four-view examination

**DOI:** 10.1186/s13089-015-0027-3

**Published:** 2015-06-12

**Authors:** Robert Arntfield, Jacob Pace, Shelley McLeod, Jeff Granton, Ahmed Hegazy, Lorelei Lingard

**Affiliations:** Western University, London, ON Canada

**Keywords:** Transesophageal echocardiography, Echocardiography, Ultrasound, Point-of-care ultrasound, Education, Simulation

## Abstract

**Background:**

Transesophageal echocardiography (TEE) offers several advantages over *transthoracic* echocardiography (TTE). Despite these advantages, use of TEE by emergency physicians (EPs) remains rare, as no focused TEE protocol for emergency department (ED) use has been defined nor have methods of training been described.

**Objective:**

This study aims to develop a focused TEE examination tailored for the ED and to evaluate TEE skill acquisition and retention by TEE-naïve EPs following a focused 4-h curriculum.

**Methods:**

Academic EPs were invited to participate in a 4-h didactic and simulation-based workshop. The seminar emphasized TEE principles and views obtained from four vantage points. Following the training, participants engaged in an assessment of their abilities to carry out a focused TEE on a high-fidelity simulator. A 6-week follow-up session assessed skill retention.

**Results:**

Fourteen EPs participated in this study. Immediately following the seminar, 14 (100 %; *k* = 1.0) and 10 (71.4 %, *k* = 0.65) successfully obtained an acceptable mid-esophageal four-chamber and mid-esophageal long-axis view. Eleven (78.6 %, *k* = 1.0) participants were able to successfully obtain an acceptable transgastric short-axis view, and 11 (78.6 %, *k* = 1.0) EPs successfully obtained a bicaval view. Twelve participants engaged in a 6-week retention assessment, which revealed acceptable images and inter-rater agreement as follows: mid-esophageal four-chamber, 12 (100 %; *k* = 0.92); mid-esophageal long axis, 12 (100 %, *k* = 0.67); transgastric short-axis, 11 (91.7 %, *k* = 1.0); and bicaval view, 11 (91.7 %, *k* = 1.0).

**Conclusion:**

This study has illustrated that EPs can successfully perform this focused TEE protocol after a 4-h workshop with retention of these skills at 6 weeks.

**Electronic supplementary material:**

The online version of this article (doi:10.1186/s13089-015-0027-3) contains supplementary material, which is available to authorized users.

## Background

Emergency physicians (EPs) frequently care for critically ill patients with acute circulatory failure or cardiac arrest. As part of this care, EPs frequently employ transthoracic echocardiography (TTE) to assist in diagnosis, therapy, and prognosis. Common and accepted applications of TTE by EPs include assessment for hemodynamically significant pericardial effusion [1–3], determining prognosis in the setting of cardiac arrest [4–6] and assessing gross left ventricular (LV) function [7–9]. Despite these important and widely used applications, acquisition of adequate TTE images can be suboptimal in up to 50 % of critically ill patients due to interference from the lungs, mechanical ventilation, surgical dressings, or patient body habitus [10, 11].

Transesophageal echocardiography (TEE) offers several advantages over TTE in the management of critically ill patients. Unlike TTE, TEE reliably obtains high-quality images in nearly all circumstances due to the probe’s indwelling esophageal location, millimeters behind the heart. This difference in acquisition and image quality has been shown to result in clinically important results with dramatically discordant success rates (97 % for TEE and 38 % for TTE) in answering clinical questions in a critically ill population [11]. TEE also has a unique role in cardiac arrest resuscitation due to its ability to interrogate the heart without interrupting chest compressions [12–15].

In spite of TEE’s superior performance, TTE is the main method EPs use to image the heart. TEE use in the emergency department (ED) is rare, with a lone small case series describing its use by a single physician [12]. Given the evidence of superior performance in critically ill patients along with the advent of TEE-compatible portable ultrasound machines and high-fidelity simulators for training, broad dissemination of TEE training to EPs is now a realistic consideration. The first steps required to effectively introduce this unique ultrasound technique to the ED are to define a focused TEE exam tailored for emergency physicians and establish acceptable methods of training for EPs.

In this study, we describe the first TEE protocol tailored specifically for the ED and evaluate the capacity of EPs to acquire the technical skills and demonstrate this protocol, on a simulator, after a 4-h training period.

## Methods

Academic emergency physicians and senior residents who routinely utilize point-of-care ultrasound independently in their clinical practice were invited to participate in a 4-h integrated didactic and simulation-based workshop on focused TEE for use in ED resuscitation. The study protocol was approved by the Health Sciences Research Ethics Board at Western University.

Potential participants were identified within our institution based on previous expressed interested in point-of-care ultrasound, including completion of advanced ultrasound training, involvement in US education, and active involvement in US research. Table 1 outlines participant demographics. Of note, 12 (85.7 %) participants had completed an advanced US course prior to study commencement.

**Table 1:** Participant demographics and characteristics

Characteristic	No. (and %) of responses
Age	
25–34	4 (28.6)
35–44	5 (35.7)
45–54	5 (35.7)
Gender	
Male	12 (85.7)
Female	2 (14.3)
Years as staff	
Resident	2 (14.3)
0–4	4 (28.6)
5–9	1 (7.1)
10–14	3 (21.4)
15+	4 (28.6)
CEUS independent practitioner status	12 (85.7)
Completion of advanced US course	12 (85.7)

*EM* emergency medicine, *CEUS* Canadian Emergency Ultrasound Society, *US* ultrasound

Two weeks prior to the workshop, participants were provided with optional supplemental reading material as recommended by the curriculum director (RA). These resources included access to a free online TEE simulator (www.pie.med.utoronto.ca) and videos demonstrating TEE examinations (see e-supplement Additional files 1 and 2).

The focused scanning protocol was devised by the curriculum director (RA) and designed to address the common scope of ED cardiac ultrasound. Recent interdisciplinary agreement over the scope of focused cardiac ultrasound in the ED has been defined to include assessment of qualitative global left ventricular function, assessment of global right ventricular size and function, assessment of the pericardial space for effusion, evaluation of volume status, and guidance of procedures (pericardiocentesis and transvenous pacemaker) [16].

**Fig. 1:**
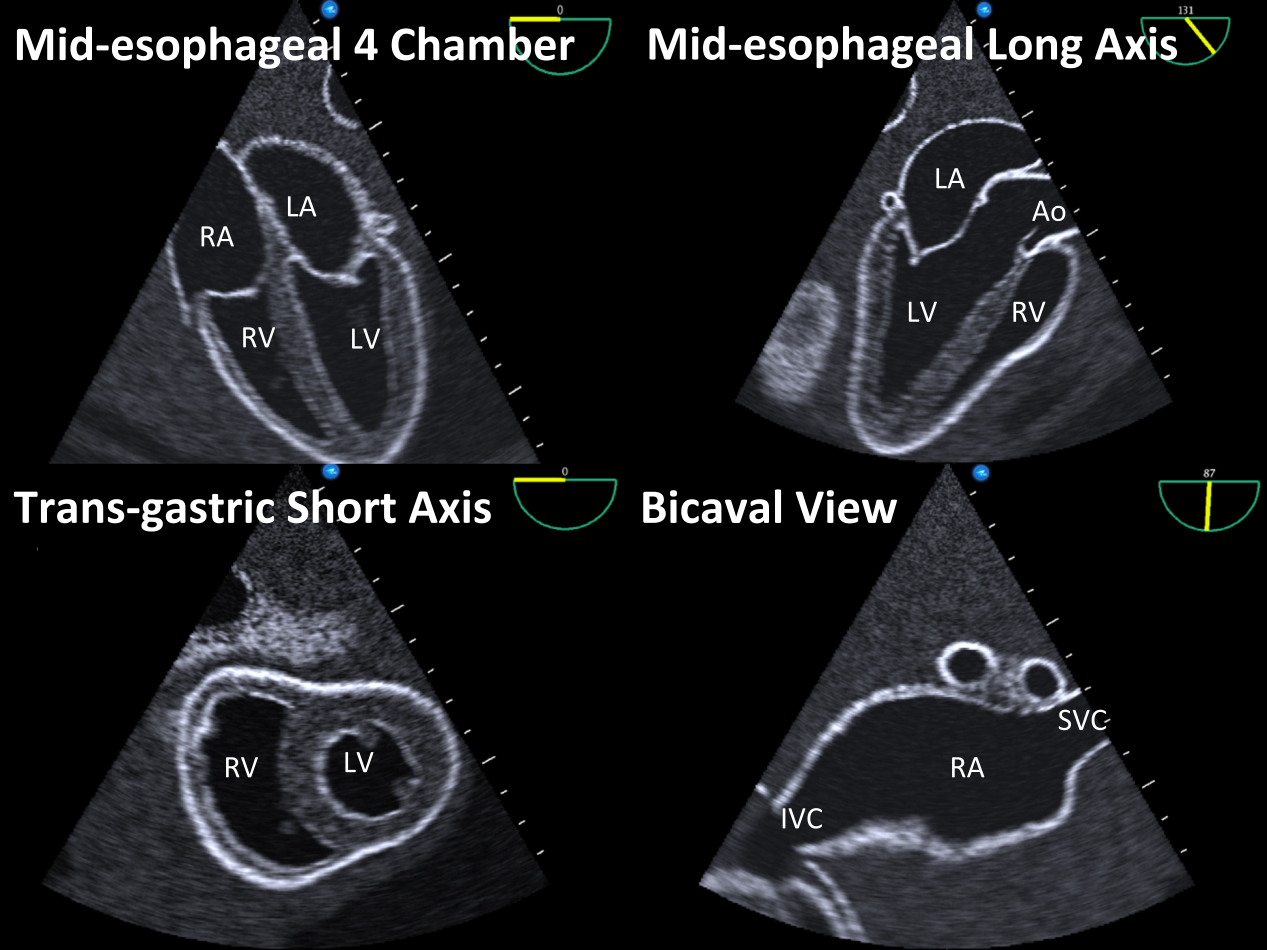
Simulator images of the four views comprising the focused TEE protocol. *RA* right atrium, *LA* left atrium, *RV* right ventricle, *LV* left ventricle, *Ao* aorta, *SVC* superior vena cava, *IVC* inferior vena cava

A comprehensive, diagnostic TEE exam consists of 28 views [17]. In assembling our protocol, we excluded 20 views from the comprehensive exam for exceeding the diagnostic goals of the EP who performs cardiac ultrasound, including detailed assessment of the thoracic aorta (6 views), assessment of segmental or quantitative left ventricular function (5 views), advanced valvular assessment (5 views), evaluation of diastolic function (3 views), and assessment of the left atrial appendage (1 view). Among the remaining eight views, the four views that we included were selected for their ability to efficiently capture the scope of ED cardiac ultrasound outlined above. The four views contained in our focused TEE protocol—mid-esophageal four-chamber view, mid-esophageal long-axis view, transgastric short-axis view, and bicaval view (Fig. 1, Table 2)—were chosen to uphold the diagnostic and procedural scope relevant to ED resuscitation.

**Table 2:** Four views of the focused TEE protocol

View	Location	Transducer controls	Structures of interest	TTE equivalent	Questions answered
Mid-esophageal four-chamber view	Mid-esophagus	0°, neutral flexion	All chambers, valves, and pericardium	Apical four-chamber	Left and right ventricular function, mitral/tricuspid valve lesions, pericardial effusion
Mid-esophageal long axis with and without color	Mid-esophagus	110–120°, neutral flexion	Left ventricle, mitral valve, aortic valve, pericardium, left atrium	Parasternal long axis with and without color	Left ventricular function, catastrophic mitral/aortic valve lesion, pericardial effusion
Transgastric short axis	Mid-stomach	0°, anteflexed	Left ventricle	Parasternal short axis	Left ventricular function, pericardial effusion
Bicaval view with M-mode	Mid-esophagus	90–100°, neutral flexion	Superior vena cava, inferior vena cava, right atrium	Subcostal IVC	Hypovolemia/volume responsiveness, procedural guidance

The workshop was taught by an emergency physician (RA), with significant echocardiographic experience in both TTE and TEE and a testamur with the National Board of Echocardiography. The workshop was composed of 2 h of lecture and 2 h of hands-on, simulated training. The lecture portion reviewed introductory TEE principles and demonstrated basic two-dimensional echocardiographic views obtained from the four standardized views outlined above.

The lecture also devoted time to image interpretation skills where, in a group format, participants practiced identifying views, anatomy, and findings on a slide set of 25 different TEE video loops. Through the use of a high-fidelity TEE simulator (Vimedix, CAE Inc, Montreal, Canada), participants engaged in a proctored image acquisition sequence. Participants also received independent guidance on TEE probe (Fujifilm Sonosite Inc, Bothell, WA) insertion on airway mannequins (Laerdal, Wappingers Falls, NY) to complement the absence of haptic simulation in the CAE simulator.

Immediately following the training, each participant underwent an assessment of their abilities to perform each of the four focused TEE views on the simulator. Time of study, used to evaluate IQR, was determined from probe insertion to the acquisition of the fourth and final view in the exam sequence. Video loops generated by participants for each view were recorded for a subsequent grading of acceptability. Grading of the views as acceptable or unacceptable was performed by three advanced echocardiographers. The curriculum director and two separate faculty (JG and AH, both of whom have completed PTEeXAM certification training in TEE) performed the quality review in an independent fashion, each being blinded to their peers’ assessments. All views were graded by two reviewers as acceptable or unacceptable; if disagreement occurred, a third reviewer graded the clip to obtain consensus.

Acceptability of images was determined by adequate depiction of the relevant anatomic features that typify each standard view and that permit accurate interpretation. Reviewers rated images based on their own expert appreciation of each view and were not prepared or trained by the principal authors.

Throughout the skill assessment, participants were asked to identify the cardiac structures demonstrated on each cardiac view. The simulator also generated various pathological states (pericardial effusion, cardiac standstill, right heart strain), and participants were asked to interpret the images generated throughout their skill assessment.

Evaluation of skill retention from this 4-h workshop took the form of a repeat simulator-based assessment 6 weeks after the original training. Participants were asked to produce each of the four views demonstrated 6 weeks earlier. Using identical assessment methods to the original workshop, all views were recorded and rated in a blinded fashion by the three reviewers as acceptable or unacceptable. Participants were free to study for this assessment as they desired but received no additional in-person instruction or access to the TEE simulator since the original workshop.

Participants were surveyed at each interval of the project (pre-workshop, post-workshop, and immediately prior to 6-week retention analysis), in order to understand their opinions on the potential application of TEE, perceived barriers to use of TEE in the ED, comfort with the modality, and any in vivo usage of TEE between the initial workshop and 6 weeks. Surveys were developed through consensus within the research team. Multiple pilot surveys were completed to ensure clarity and comprehension.

Data were entered directly into a study-specific spreadsheet. Descriptive statistics were summarized using means and standard deviations or proportional differences with 95 % confidence intervals where appropriate. Statistical analysis was conducted using Stata 13.0 (StataCorp LP, College Station, TX). Inter-rater reliability was estimated using multirater kappa (*k*) statistics, with *k* = 0.61–0.80 interpreted as “good agreement” and *k* > 0.80 interpreted as “very good agreement” [18].

## Results

A convenience sample of 14 EPs experienced in point-of-care ultrasound, including basic TTE, consented to participate in the study. Participants were predominantly male but otherwise represented varying ages and career stages (Table 1). All participants reported already incorporating transthoracic cardiac ultrasound into cardiac arrest management, and all “agreed” or “strongly agreed” that TEE would be beneficial during cardiac arrest resuscitation. The greatest perceived barriers to TEE use in the ED included “access to TEE probe,” “resource and time allocation,” and “concern with ability to obtain views” (Table 3).

**Table 3:** Participant-perceived barriers to transesophageal echocardiography in the emergency department

Barrier	No. (and %) of responses	
	Pre-workshop (*n* = 14)	Post-workshop (*n* = 12)
Access to TEE probe	13 (92.9)	9 (75.0)
Resource and time allocation	6 (42.9)	7 (58.3)
Ability to acquire views	6 (42.9)	5 (41.7)
Complications secondary to probe insertion	4 (28.6)	1 (8.3)
Personal apprehension	1 (7.1)	1 (8.3)

The post-workshop survey revealed that all study participants were “satisfied” or “extremely satisfied” with the educational session. Seven participants suggested increasing duration of hands on simulation as a means to enrich the experience. No other deficiencies were identified. Thirteen participants reviewed the supplemental pre-course material, and 12 reported an increase in confidence with TEE and overall workshop experience as a result of reviewing the material.

Immediately following the workshop, 12 (85.7 %) participants reported feeling confident in their ability to use the training to acquire clinically acceptable TEE views in vivo. The results of the simulator-based, post-workshop skills assessment are summarized in Table 4. Acceptable image acquisition and inter-rater agreement were as follows: mid-esophageal four-chamber, 14 (100 %; *k* = 1.0); mid-esophageal long axis, 10 (71.4 %, *k* = 0.65; 95 % CI 0.21, 1.0); transgastric short-axis, 11 (78.6 %, *k* = 1.0); and bicaval view, 11 (78.6 %, *k* = 1.0) for a total success in 82.1 % of potential views (*k* = 0.88; 95 % CI 0.71, 1.0). Median (IQR) time to study completion was 2.2 (1.6, 3.4) minutes. Nine of 14 (64.3 %) participants subsequently used TEE for the first time in patient care after the original workshop and prior to the 6-week retention assessment.

**Table 4:** Number of participants achieving successful transesophageal echocardiography image acquisition

Cardiac view	Number (and %) of participants achieving successful image acquisition	
	Post-workshop (*n* = 14 participants)	6-week retention (*n* = 12 participants)
Mid-esophageal four-chamber	14 (100 %), *k* = 1.0	12 (100 %), *k* = 0.92 (95 % CI 0.76, 1.0)
Mid-esophageal long axis	10 (71.4 %), *k* = 0.65 (95 % CI 0.21, 1.0)	12 (100 %), *k* = 0.67 (95 % CI 0.39, 0.95)
Transgastric short-axis	11 (78.6 %), *k* = 1.0	11 (91.7 %), *k* = 1.0
Bicaval	11 (78.6 %), *k* = 1.0	11 (91.7 %), *k* = 1.0
Totals	46 (82.1 %), *k* =	46 (95.8 %), *k* =

Twelve participants engaged in a skill retention assessment 6 weeks post-workshop with 2 of the original 14 participants unable to attend due to scheduling conflicts. Prior to assessment, 8 of 12 (66.7 %) participants reported feeling “confident” or “very confident” in their ability to acquire the four cardiac views. Simulation-based skills retention assessment revealed acceptable images and inter-rater agreement as follows: mid-esophageal four-chamber, 12 (100 %; *k* = 0.92; 95 % CI 0.76,1.0); mid-esophageal long axis, 12 (100 %, *k* = 0.67; 95 % CI 0.39, 0.95); transgastric short-axis, 11 (91.7 %, *k* = 1.0); and bicaval view, 11 (91.7 %, *k* = 1.0) for an overall success rate of 95.8 % possible views (*k* = 0.79; 95 % CI 0.62, 0.96) (Table 4). Median (IQR) time to study completion was 3.0 (2.4, 3.2) minutes.

During both the immediate skill assessment and the 6-week retention assessment, all participants were able to accurately identify both cardiac structures on the acquired views. Pathological conditions were correctly identified by 100 % of participants during both the immediate and 6-week skill assessment.

## Discussion

TEE is commonly indicated for and typically regarded as a tool for advanced diagnostic questions (e.g., ruling out endocarditis or assessment of the left atrial appendage for clot prior to cardioversion) that may rarely be of interest to the point-of-care sonographer carrying out a more focused exam. Due to its unique properties, however, TEE has also shown to be a valuable tool in caring for the critically ill. Its indwelling nature permits continuous acquisition to monitor cardiac responses to interventions such as fluids, vasoactive medications, and CPR. Additionally, TEE provides higher resolution images compared to TTE while also being less vulnerable to inter-user variability or patient factors, such as ventilation or obesity, due to its predictable retrocardiac imaging location [19]. In anticipation of studying and understanding how these properties may benefit frontline ED resuscitation, we have developed a focused TEE scanning protocol and a corresponding curriculum for its introduction to EPs. We have shown that EPs who attend a 4-h TEE workshop can acquire the cognitive and motor skills necessary to generate four focused TEE views, both immediately after and 6 weeks after training, with very good success rates on a high-fidelity simulator.

In instructing EPs to apply this TEE protocol, high levels of success with acquisition of the four views were found in the majority of participants in both the immediate post-workshop evaluation (82.1 % views successful) and the retention analysis at 6 weeks (95.8 % views successful, Δ 13.7 %, 95 % CI 1.1 %, 26.0 %). This significantly improved overall performance at 6 weeks runs contrary to typical results from similar simulation research [20, 21]. We are unable to entirely explain this finding but may be related to the duration of time elapsed between initial training and the retention analysis. Ideal timing for skill retention testing is unclear, and recent article on simulation education methods was unable to establish recommended time frames for such retention assessments [22]. It is plausible that the excellent skill retention demonstrated in our study was influenced by an insufficient time lapse between initial workshop and retention analysis, thus not capturing skill decay occurring over larger time intervals. The absence of two participants who did not complete retention analysis, who had excellent performance in the initial skills analysis, are an unlikely source for relative improvement. It may be speculated that, as our participants were ultrasound credentialed already, that the absence of skill decay may be explained by TEE being a minor procedural variance on these existing skills rather than being learned as a new task.

The mid-esophageal four-chamber view was acquired successfully at both intervals by all participants. As the “default” view one acquires from the mid-esophagus, it requires the least amount of probe manipulation and is, in general, easiest to obtain. The other views, however, all require some degree of probe manipulation. Probe advancement and/or flexion is required for the transgastric short axis, multiplane angle adjustment for the mid-esophageal long-axis, and probe rotation with multiplane angle adjustment for the bicaval view. Due to the intricacy and novelty of these movements, these views were expectedly more challenging for users, with slightly lower success rates on average, immediately after the training session (Table 4).

Seven of 14 participants suggested that the educational experience could be improved by increasing the duration of time spent using the simulator to practice image acquisition. Increasing resources either through expanded duration of the workshop or deploying multiple simulators would be most logical. The overall strong results by the participants demonstrating initial and retained TEE procedural skills suggest that limited simulator time may be less of an influence on training and more of an influence on the subjective learning experience. Traditional TEE users, such as cardiologists or cardiac anesthesiologists, may devote upwards of 1 year of training to achieve competency in TEE.

The minimally invasive nature of TEE poses significant challenges for the widespread dissemination of the modality. Traditional methods of instructing point-of-care ultrasound that rely heavily on the use of human volunteers during initial coursework and acquisition training are not possible with TEE. The advent of high-fidelity TEE simulators has now enabled new methods of training to be considered while also providing access to hands on acquisition training for those providers, such as EPs in our study, without routine access to patient care environments where TEE and its training are carried out. The value of simulators extends beyond providing training opportunities for those who are TEE-naïve and has recently been the subject of study by cardiologists and cardiac anesthesiologists. In two separate studies, educators from both disciplines found that adding simulation to traditional clinical training improved learner outcomes [23, 24].

As no TEE simulator yet mimics the haptics of TEE probe insertion, we elected to devote separate training for this portion of the procedure using standard airway mannequins and actual TEE probes, asking participants to intubate the esophagus several times. In experienced hands, mechanical complication rates of TEE examinations are reported to be very low at 0.18 % [25]; however, oropharyngeal injury or esophageal injury is a genuine concern of both newcomers to TEE and traditional users alike. Simulating probe insertion was intended to build comfort among TEE operators prior to using the tool in vivo and minimize risk of oropharyngeal injury among patients.

### Limitations

Our study size was limited by the number of TEE probes (three) and simulators (one) at our institution. Our study population may have suffered from selection bias as physicians were invited to participate based on previous experience with point-of-care ultrasound. Unfortunately, the haptics of esophageal intubation are difficult to reproduce with a simulator; we attempted to resolve this issue by demonstrating esophageal intubation on airway mannequins using TEE probes. This is a limitation commonly encountered in procedural simulation and will hopefully resolve as simulation fidelity improves.

Despite the small size and heterogeneity of our study population, we anticipate that the structure and strong success of our TEE curriculum will provide the first and most important step towards the broader dissemination of TEE in the ED for resuscitation, particularly when TTE is indeterminate or insufficient to resolve pressing questions related to cardiac structure or function.

## Conclusions

Emergency physicians with experience in point-of-care ultrasound are able perform a four-view, focused TEE exam with high success using a simulator, after 4 h of training with retention of these skills at 6 weeks. This is the first focused TEE scanning protocol for emergency physicians and may serve as a model for widespread dissemination and training.

## Additional files

Additional file 1:
**Probe handle controls.**
Additional file 2:
**Demonstrated clinical exam.**
